# Anti-Ganglioside Antibodies in Amyotrophic Lateral Sclerosis Revisited

**DOI:** 10.1371/journal.pone.0125339

**Published:** 2015-04-14

**Authors:** Katja Kollewe, Ulrich Wurster, Thomas Sinzenich, Sonja Körner, Reinhard Dengler, Bahram Mohammadi, Susanne Petri

**Affiliations:** 1 Department of Neurology, Medical School Hannover, Hannover, Germany; 2 Department of Neurology, University of Lübeck, Lübeck, Germany; 3 CNS-LAB, International Neuroscience Institute, Hannover, Germany; University of Jaén, SPAIN

## Abstract

**Background:**

Amyotrophic Lateral Sclerosis (ALS) is a devastating neurodegenerative disorder with typical onset in the 5^th^- 6^th^ decade of life. The hypothesis of an autoimmune origin of ALS receives less attention today, but immunological phenomena still seem to be involved and mechanisms such as protective autoimmunity may be important. Detection of antibodies against a variety of gangliosides has been repeatedly described in ALS-patients by several authors, but widely differing frequencies and titres have been reported. Therefore, we investigated the presence of six common antibodies with a commercially available test panel for GA1, GM1, GM2, GD1a, GD1b and GQ1b in a large group of clinically well-characterized ALS patients and compared them to a collective of 200 healthy blood donors.

**Methods:**

IgG and IgM antibodies to the six gangliosides asialoGM1 (GA1), GM1, GM2, GD1a, GD1b, GQ1b were determined by GanglioCombi ELISA in sera of 84 ALS patients. Results were expressed as a %-ratio of a highly positive control and categorized as negative (<30%), borderline (30–50%), moderately (50–100%) and strongly positive (>100%). The values obtained from 200 Swiss blood donors served as a reference group.

**Results:**

In twenty-two (26.2%) ALS-patients elevated anti-ganglioside antibodies could be detected: Taking all subspecific antibodies together, IgG antibodies were found in 9/84 (10.7%) and IgM in 15/84 (17.9%) patients. There was no correlation between age, gender, site of onset or survival and anti-ganglioside-positive/-negative titres in ALS-patients. No statistically significant difference in the frequency of anti-ganglioside antibodies compared to the group of healthy blood donors was found.

**Conclusion:**

Even with this more comprehensive approach, anti-ganglioside antibody frequencies and patterns in our ALS cohort closely resembled the values measured in healthy controls. In accordance with other studies, we did not observe any association of a distinct ALS phenotype with elevated anti-ganglioside antibodies or an impact on survival.

## Introduction

Amyotrophic Lateral Sclerosis (ALS) is a devastating neurodegenerative disorder with typical onset in the 5^th^- 6^th^ decade of life. Selective loss of motor neurons in the primary motor cortex, brainstem and spinal cord results in rapidly progressive paralysis of bulbar, limb and voluntary muscles. Death usually occurs within 3–5 years after diagnosis, mostly due to respiratory failure [1–3]. The diagnosis of ALS can be difficult in early disease stages, especially when symptoms are limited to the lower motor neuron. In these cases, it is crucial to distinguish ALS from multifocal motor neuropathy (MMN). As opposed to ALS, MMN responds to immunomodulatory treatment with intravenous immunoglobulins and does in general not decrease life expectancy. High anti-ganglioside antibody titres, particularly GM1-specific IgM antibodies, can be detected in approximately 50% of patients with MMN [4–6]. The reported prevalence of anti-GM1 antibodies in MMN varies widely between 20% and 85%, particularly due to methodological differences [4;6–9]. However, antibodies against a variety of gangliosides with widely differing frequencies and titres have also been described in ALS [4;10–16]. It has been hypothesized that anti-ganglioside antibodies may play a pathogenic role in ALS as gangliosides are known to be involved in neuronal development and regeneration [17]. Gangliosides are glycosphingolipids located on plasma membranes throughout the body. In neural tissues, higher concentrations can be found [18]. Physiological functions of gangliosides postulated so far include modulation of membrane proteins, neural development and differentiation, cell-cell interaction and adhesion, neuronal Ca^++^ homeostasis, temperature adaptation, axonal growth, (para)node of Ranvier stability and synaptic transmission [19–23]. They also contribute to the regulation of several receptors, such as neurotrophic factor, neurotransmitter, muscarinic acetylcholine, serotonin, glutaminic acid, and complement regulatory protein receptors [23;24]. Different nervous system structures express different ganglioside expression patterns and levels: GM1-gangliosides occur at higher concentration in ventral compared to dorsal root nerve myelin [25], bind to the surface of spinal cord neurons but not to sensory ganglia neurons [26] and are preferentially exposed only on the surface of the myelinated fibers at the paranodal region [27]. GD1a-ganglioside is more concentrated in motor than in sensory nerves and more in axons than in the myelin [25;28]. GM1 and GD1a are enriched at paranodal regions of nodes of Ranvier in myelinated peripheral nerve axons [20;29;30]. GD1b-gangliosides have been found to be enriched in cranial motor nerves supplying the extraocular muscles [31]. High GQ1b expression is found selectively at paranodal regions in cranial nerves with oculomotor function [32;33].

Antibodies against these molecules can be found in a variety of neurological diseases such as Alzheimer’s disease [34], Parkinson’s disease [35;36], Multiple Sclerosis [37], systemic lupus erythematosus [38], peripheral neuropathy [4], chronic inflammatory demyelinating polyneuropathy [39], Guillain-Barré-syndrome (GBS) [40–43], Miller Fisher syndrome [44] and multifocal motor neuropathy (MMN) [6;45;46].

The frequency and pathogenic role of anti-ganglioside antibodies in ALS has been controversially described and discussed in the literature. Some authors have postulated an association between the presence of anti-ganglioside antibodies and predominant lower motor neuron involvement [10;16;47], whereas others have not found this correlation [11;48;49].

While these studies have mainly tested antibodies against gangliosides GM1, we have now investigated a much wider spectrum including the most frequent and clinically relevant antibodies against GA1, GM1, GM2, GD1a, GD1b and GQ1b in a large group of clinically well-characterized ALS patients and their potential correlation with different clinical phenotypes and survival times and compared them to a group of 200 healthy blood donors.

## Patients and Methods

Sera from 84 ALS patients (mean age 58 years, range 28–81 years, 45 men, 39 women) were included in the analysis. All patients fulfilled the diagnostic criteria for probable or definite ALS according to the revised El Escorial criteria of the World Federation of Neurology during the course of the disease [50] and have been regularly followed for at least 4 times in our ALS-outpatient-clinic in three to four months intervals. Sera of these patients were collected once when they were diagnosed with ALS and stored at -70°C until analysis. The amyotrophic lateral sclerosis functional rating scale revised (ALSFRS-R) [51] was used to assess the severity and progression of the disease. Disease progression was calculated by dividing the difference between ALSFRS-R score at the first visit (time of sample collection) and the ALSFRS-R score at the last visit by the time (in months) between the two visits. Survival was defined as time from symptom onset until death. The values obtained from 200 healthy blood donors (mean age 52 years, range 18–70 years, 118 men, 82 women) served as a reference group. Upon admission at Hannover Medical School, patients had signed a general informed consent form agreeing to anonymized use of their clinical data and analysis of body fluids for scientific purposes. The data were analysed anonymously. The ethics committee at Hannover Medical School approved the study.

### Anti-ganglioside antibody examinations

Samples were investigated by enzyme-linked immunosorbent assay (ELISA) technique (BÜHLMANN GanglioCombi(**R**) ELISA, BÜHLMANN Laboratories AG, Schönenbuch, Switzerland), for antibodies against GM1, GA1, GM2, GD1a, GD1b and GQ1b. Briefly, the 6 gangliosides are pre-coated onto microtiter plates. Serum samples as well as calibrator and controls are incubated for two hours at 2–4°C. Unbound antibodies are washed away. An enzyme labelled anti-human antibody against either IgG or IgM is added and incubated for another 2 hours at 2–4°C. Unbound secondary antibodies are washed away and a colour substrate (TMB) is added. After an incubation time of 30 minutes at room temperature, the reaction is stopped and the OD is measured at 450 nm.

The colour intensity is directly proportional to the amount of bound antibody. Results (which are expressed as % ratio) are attributed to one of the clinically validated titre categories (negative (<30%), greyzone (30–50%), positive (50–100%), strongly positive (>100%); cut-off = 50%). For further statistical analysis, differentiation was limited to negative (<30%) or positive (>30%) results.

### Statistical analysis

Demographic data and antibody positivity rates of the patient and healthy control groups were compared using Student’s t-test. A p-value <0.05 was considered as statistically significant. The following parameters and their relation to survival were studied: age, gender, site of onset, presence/absence of anti-ganglioside antibodies. The influence of these parameters on survival was studied using the Kaplan-Meier life table methods, Log rank test was used to assess the significance. Statistical analyses were done using the SPSS v. 17 (SPSS, Chicago, IL) program.

## Results

### Patients

At time of analysis, 65 patients were deceased, 19 were still alive, and no patient was lost to follow-up.

Patients were divided into a group with (n = 22) and without elevated anti-ganglioside antibodies (n = 62) and further analysis regarding age, gender, site of onset (bulbar- or limb-onset), ALSFRS-R at time of sample-collection, time between symptom-onset and sample-collection (equivalent to time point of diagnosis) and survival was performed (Table 1). There was no statistically significant difference between the two groups regarding the above mentioned parameters. Monoclonal proteins were present in 5 out of 84 patients (5.9%; three times IgG, two times IgM, all in patients without elevated anti-ganglioside antibodies) and thus within the reported normal range for the age-standardized prevalence in Germany [52].

**Table 1:** Data regarding age, gender, site of onset, ALSFRS-R at time of sample-collection, disease progression and survival in ALS-patients with and without elevated anti-ganglioside antibodies.

	ALS-patients with elevated anti-ganglioside antibodies (n = 22)	ALS-patients without anti-ganglioside antibodies (n = 62)
Age (years)	57.4 (SD = 13.9)	57.9 (SD = 9.8)
Gender (male/female)	12 (54.5%)/10 (45.5%)	33 (53.2%)/29 (46.8%)
Bulbar-onset	6 (27.3%)	16 (25.8%)
Limb-onset	16 (72.7%)	46 (74.2%)
Survival (months) form symptom onset	46.5 (SD = 31.9)	43.3 (SD = 22.4)
ALSFRS-R at time of sample-collection	42.5 (SD = 4.0)	42.6 (SD = 3.6)
Disease progression (ALSFRS-R-ratio)	0.71 (SD = 0.48)	0.77 (SD = 0.52)
Time (months) between symptom-onset and sample-collection	17.1 (SD = 22.9)	16.2 (SD = 15.0)

The 4 patients with strongly positive IgG or IgM anti-ganglioside antibodies showed no particular clinical phenotype: the mean age was 57.8 years, 2 had bulbar-onset and were women, 2 had limb-onset (one man, one woman). None of these patients showed a lower motor neuron syndrome. All 4 patients presented with clinical and neurophysiological signs of upper and lower motor neuron involvement. There was no evidence for abnormalities in nerve conduction velocities in all 4 patients. They were all treated with Riluzole; one patient additionally received 2 cycles of intravenous immunoglobulin, although she had upper motor neuron involvement, but the disease was progressive under this therapy and it was therefore terminated. The 2 patients with bulbar onset died after 18 and 21 months, respectively, the 2 patients with limb-onset were still alive 4 years after symptom-onset; the mean ALSFRS-R of these 4 patients at time of sample-collection was 43.5.

As previously shown in our cohort [1], gender showed no effect on survival. However, age at onset and bulbar-onset was an independent prognostic factor in our population (log-rank p < 0.01).

### Anti-ganglioside antibody examinations

#### Anti-ganglioside antibodies in ALS-patients

Taking all subspecific antibodies together, IgG antibodies were found in 9/84 (10.7%) and IgM in 15/84 (17.9%) patients (Table 2). As 2/84 (2.4%) patients (no. 18 and no. 20) exhibited both isotypes IgG and IgM, the combined frequency is 22/84 (26.2%). Three simultaneous antibodies occurred in 1 patient each for IgG (no. 22, GA1, GD1a, GQ1b) and IgM (no. 13, GM1, GD1a, GD1b). Two simultaneous antibodies occurred in 1 patient each for IgG (no. 20, GM2 and GD1a) and IgM (no. 19, GA1 and GM1). Strongly positive values were found in 4 (2 in IgG and 2 in IgM) out of 84 (4.8%). However, the frequencies for the individual antibodies were rather low, with maximal 7.1% (6/84) for IgG GD1a and 8.3% (7/84) for IgM GA1.

**Table 2:** Data of 22 ALS-patients with greyzone (*), positive (^#^) and strongly positive (^§^) results (expressed as % ratio of Calibrator).

	IgG						IgM					
	GA1	GM1	GM2	GD1a	GD1b	GQ1b	GA1	GM1	GM2	GD1a	GD1b	GQ1b
1	3	4	3	4	4	4	12	42*	18	4	4	3
2	3	3	2	41*	7	24	5	4	2	6	4	2
3	3	3	3	4	3	4	19	15	14	5	7	36*
4	3	3	2	26	2	24	10	49*	3	10	5	23
5	4	4	3	11	3	3	3	35*	2	5	7	4
6	3	5	2	5	4	6	23	18	8	45*	22	5
7	3	2	2	8	2	17	41*	10	3	5	3	4
8	4	30*	2	8	2	3	3	3	2	3	3	2
9	3	4	2	4	3	3	11	12	10	4	39*	3
10	3	3	2	4	2	3	32*	8	5	6	8	8
11	3	3	3	75^#^	4	5	6	6	6	4	4	5
12	4	3	2	87^#^	2	8	11	6	3	6	4	6
13	7	4	9	8	27	8	42*	27	11	31*	71^#^	5
14	4	3	3	3	3	4	57^#^	22	5	22	25	6
15	3	3	2	4	2	4	55^#^	27	10	6	11	8
16	90^#^	6	4	12	20	8	4	6	2	3	3	7
17	2	2	2	23	70^#^	6	13	23	3	4	4	3
18	3	3	3	73^#^	4	6	48*	18	11	7	17	7
19	6	8	5	5	7	4	132^§^	51^#^	26	6	13	13
20	3	4	36*	262^§^	16	5	10	14	2	47*	15	3
21	3	3	2	4	3	3	5	107^§^	3	4	5	3
22	110^§^	3	2	198^§^	3	68^#^	6	5	2	4	3	5

#### Anti-ganglioside antibodies in healthy blood donors

IgG antibodies were found in 31/200 (15.5%) and IgM antibodies in 29/200 (14.5%). In 5 out of 200 (2.5%) controls both isotypes (IgG and IgM) could be found. Therefore, the combined frequency is 55/200 (27.5%). Strongly positive values were found in 7 (6 in IgG and 1 in IgM) out of 200 (3.5%) normal blood donors. Slightly increased IgM-GA1 antibody titres were detected in 8%.

Anti-ganglioside antibody frequencies and patterns in healthy blood donors closely resembled the values observed in ALS-patients. There was no statistically significant difference to the collective of ALS patients.

### Statistical analysis

There was no correlation between age, gender, site of onset, time between symptom-onset and diagnosis or survival and presence or absence of anti-ganglioside antibodies in ALS-patients. There was no statistically significant difference in the disease progression between ALS-patients with (mean: 0.71; SD = 0.48) and without elevated anti-ganglioside antibodies (mean: 0.77; SD = 0.52; p = 0.6). To check if there were any correlations between disease severity or disease progression with the titres of anti-ganglioside antibodies, we tested the ALSFRS-R-score at the time of sample collection and its ratio (as described above) against anti-ganglioside antibodies using regression analysis. There were no significant correlations, neither in the whole group of ALS-patients nor in ALS-patients with elevated anti-ganglioside antibodies.

## Discussion

In this study, we investigated the presence of IgG and IgM antibodies against the 6 most frequent and clinical relevant gangliosides (GM1, GA1, GM2, GD1a, GD1b and GQ1b) in 84 clinically well-characterized ALS-patients. Twenty-two ALS-patients (26.2%) had elevated anti-ganglioside antibody levels. When we compared these 22 patients with the antibody-negative group, no differences in age, gender, site of onset, clinical presentation, disease progression or survival became apparent. Also the four patients with strongly positive IgG or IgM antibodies showed no particular clinical characteristics regarding disease stage at the time of blood sampling, disease progression, presence, predominance or severity of lower motor neuron signs or abnormalities in nerve conduction velocities. Furthermore, we did not observe significant differences of anti-ganglioside antibody patterns in our ALS cohort in comparison to a control group of 200 healthy blood donors.

To the best of our knowledge, this wide variety of subspecifities of anti-ganglioside antibodies has not been investigated in ALS patients to date in such a big sample size (n = 84) and in comparison to healthy controls using the same diagnostic kit.

Antibodies against a variety of gangliosides have been reported to occur in ALS [4;14;15;45;53;54] in a wide frequency, ranging from 0% to 78% [10–12;47–49;55–58]. The detection of elevated IgM anti-ganglioside antibodies in 17.9% of the patients (5.58% anti-GM1 IgM) in our cohort therefore is in the lower range of the previously reported data. It is controversially discussed if the presence of anti-ganglioside antibodies is associated with a distinct clinical presentation/ phenotype or longer or shorter survival:

Niebroj-Dobosz and colleagues [48] found neither a correlation between the titre of anti-neural antibodies and the course of ALS, nor age, sex, site of onset, duration time and severity of the disease. They concluded that the elevated antibodies in some ALS patients occurred as manifestation of an autoimmune response of low activity, but that this was rather an epiphenomenon of neuronal degeneration.

Lamb and colleagues [11] found no significant correlations between anti-GM1 titre and the following variables: age, sex, presence of bulbar muscle involvement, onset of symptoms longer or shorter than 3 years prior to obtaining the anti-GM1 titre.

In a cohort of 43 cases with progressive lower motor neuron syndromes including motor neuropathy with conduction block, and 103 ALS-patients, no correlation between elevated titres of IgM anti-GM1 ganglioside antibodies and age, sex or disease duration was found either [49].

On the other hand, Pestronk reported that IgM anti-GM1 antibodies were more frequent in ALS-patients presenting with prominent lower motor neuron signs [10]. Other correlations with other parameters such as age or survival were not described in this study. The results were replicated in a further study of the same group one year later [47]. In this study, antibodies to GD1a gangliosides were investigated additionally to GM1 gangliosides, which led to a percentage of 78% of elevated antibodies in ALS-patients, who had mainly prominent lower motor neuron signs. The investigation of 24 normal controls (patients or volunteers without systemic disease) found only 8% of patients with elevated antibodies.

Yuki and co-workers found high titres of IgG anti-GM1 antibodies, but no IgM anti-GM1 antibodies in three out of 655 patients with suspected ALS. These patients had no upper motor neuron signs and were therefore diagnosed as lower motor neuron syndrome. The authors stated that “… both IgM and IgG antibodies should be tested in patients with lower motor neuron syndrome to discover treatable patients” [16].

Other groups [7;12;54;58–60] who investigated anti-ganglioside antibodies in ALS did not focus on the correlation of titres and clinical presentation or survival but only described percentages of antibodies in different diseases, ranging from 2% in myasthenia gravis to 90% in patients presenting with a motor neuropathy [12].

Initial investigations were limited to anti-GM1 ganglioside antibodies and showed widely differing proportions from 21% [49] to 78% [10] of ALS patients with positive antibodies. Possible explanations for the discrepant findings include purity of ganglioside preparations, incubation temperature, use of detergent, solid phase material and different cut-off values. Former in-house assays have now been largely replaced by better standardized commercial tests. Instead of serial serum dilutions, only one dilution (1:50) is applied in the assay used here. This approach is deemed appropriate for routine evaluation and helps to keep costs down. The OD (optical density) values obtained from the ELISA are converted to %-ratios by relating the OD of the respective ganglioside antibody to the OD of a calibrator and multiplied by factor 100. For example: patient no. 21 in Table 2 with a measured OD of 2.061 yielded a 107% ratio for GM1 IgM after division by 1.926, the OD of the calibrator. Results are expressed in four categories in relation to the highly reactive calibrator as negative: < 30%, grey zone: 30%- 50%, positive: > 50%- 100% and strongly positive: > 100%. For GM1 IgM antibodies OD values < 0.58 would therefore be categorized as negative, although a distinct signal is still present. However, the decision limits of the 4 categories were defined by the manufacturer after an extended multi-center study with clinically well-defined probes and due consideration of an apparently healthy control group. Titration is a semi-quantitative method and the definition of the final dilution in terms of OD is arbitrary and depends on the chosen cut-off. A direct conversion of %-ratios to titres is therefore difficult, but a rough estimation with a background OD value of 0.100 would result in a titre of approximately 1:1000 for the above case. A recent comparative evaluation of four commercially available ganglioside antibody tests (1) Immunoline Generic Assays, Dahlewitz, Germany, (2) Immunodot Dotzen-Zentec, Angleur Belgium, (3) Immunoline ganglio profile, Euroimmun, Lübeck, Germany, (4) BÜHLMANN GanglioCombi ELISA, BÜHLMANN Laboratories AG, Schönenbuch, Switzerland) still revealed considerable differences in sensitivity and reactivity [61]. The ELISA test (4) is frequently used in Europe and provides a panel of 6 different ganglioside specificities of human origin. Concordance with negative and highly positive samples was good compared to the in-house (blotting) method defined as the reference method by Caudie [61]. Comparison of antibody levels between different assays is hampered by the lack of internationally available and recognized autoantibody standards.

The present study was not intended to elucidate potential pathogenetic roles of anti-ganglioside antibodies. Although highly interesting and important such studies would have to be carried out on individual cases with high autoantibody levels. Ideally such patients should be followed longitudinally to find out whether the development of new clinical signs could be related to changes in antibody expression and distinctive patterns. The specificity of a certain anti-ganglioside antibody in vivo is far from clear and epitope sharing between glycolipids and glycoproteins may occur. In this regard the claim made by Caudie [61] that blotting membranes (PVDF) offer a superior solid phase, because the lipophilic parts of the ganglioside molecules are buried inside, whereas the antigenic carbohydrate moieties are exposed on the surface, may not reflect the actual situation in nervous tissue. Indeed, it has been shown that simultaneous presentation of two different gangliosides (ganglioside complexes) result in different antibody binding compared to single gangliosides alone [62]. Gangliosides are part of lipid rafts and adjacent molecules may form new conformational epitopes which do not exist when single antigens are coated to an artificial membrane.

## Conclusion

In the present study, we describe for the first time the utilization of a state of the art-ELISA assay to obtain reliable data on the occurrence of IgG and IgM antibodies to GA1, GM1, GM2, GD1 a, GD1b, GQ1b gangliosides in a well characterized group of 84 ALS patients.

The more comprehensive assessment did not reveal a higher percentage of ALS-patients positive for anti-ganglioside antibodies as compared to healthy controls. In accordance with previous studies, no specific ALS phenotype associated with the presence of anti-ganglioside antibodies was observed nor an influence disease progression or on survival. Anti-ganglioside antibodies are therefore not suitable as diagnostic or prognostic markers in ALS. Detection of anti-GM1 antibodies can however, in the context of corresponding clinical presentation and electrophysiological data, be helpful to support the diagnosis of MMN, an important and treatable differential-diagnosis of ALS.
